# Modulation of Functional Phosphorylation Sites by Basic Residues in the Unique Domain of c-Src

**DOI:** 10.3390/molecules28124686

**Published:** 2023-06-10

**Authors:** Andras Lang, Alejandro Fernández, Mireia Diaz-Lobo, Mar Vilanova, Francisco Cárdenas, Margarida Gairí, Miquel Pons

**Affiliations:** 1 BioNMR Laboratory, Departament de Química Inorgànica i Orgànica, Universitat de Barcelona (UB), Baldiri Reixac 10-12, 08028 Barcelona, Spain; 40lang@ub.edu (A.L.); alejandrofernandez@ub.edu (A.F.); 2 Institute for Research in Biomedicine (IRB Barcelona), The Barcelona Institute of Science and Technology (BIST), Baldiri Reixac 10, 08028 Barcelona, Spain; mireia.diaz@irbbarcelona.org (M.D.-L.); mar.vilanova@irbbarcelona.org (M.V.); 3 Centres Científics i Tecnològics de la Universitat de Barcelona (CCiTUB), Universitat de Barcelona (UB), Baldiri Reixac 10-12, 08028 Barcelona, Spain; francardenas@ub.edu (F.C.); mgairi@ub.edu (M.G.)

**Keywords:** pKa measurements, phosphoserine, phosphothreonine, guanidinium–phosphate salt bridge, fuzzy complex, intrinsically disordered proteins, IDP phosphorylation, ERK2, SNRE

## Abstract

In contrast to the well-studied canonical regulatory mechanisms, the way by which the recently discovered Src N-terminal regulatory element (SNRE) modulates Src activity is not yet well understood. Phosphorylation of serine and threonine residues modulates the charge distribution along the disordered region of the SNRE and may affect a fuzzy complex with the SH3 domain that is believed to act as an information transduction element. The pre-existing positively charged sites can interact with the newly introduced phosphate groups by modulating their acidity, introducing local conformational restrictions, or by coupling various phosphosites into a functional unit. In this paper, we use pH-dependent NMR measurements combined with single point mutations to identify the interactions of basic residues with physiologically important phosphorylated residues and to characterize the effect of these interactions in neighbor residues, thus providing insight into the electrostatic network in the isolated disordered regions and in the entire SNRE. From a methodological point of view, the linear relationships observed between the mutation-induced pKa changes of the phosphate groups of phosphoserine and phosphothreonine and the pH-induced chemical shifts of the NH groups of these residues provide a very convenient alternative to identify interacting phosphate groups without the need to introduce point mutations on specific basic residues.

## 1. Introduction

c-Src is the leading member of the Src family of kinases (SFK) and the first discovered proto-oncogene. It mediates signal transduction processes related to cell migration, invasion, and survival [1]. High levels of c-Src activity are associated with a poor prognosis in different cancers [2,3]. All members of the SFK share the same domain architecture formed by highly conserved globular Src homology domains (SH1, SH2, and SH3) and an N-terminal disordered region formed by the SH4 and Unique domains. The canonical regulatory mechanisms involving the globular domains have been extensively studied [4,5]. However, the regulation layer involving the intrinsically disordered region of c-Src [6,7] remains poorly understood. Phosphorylation of serine and threonine residues are important players in the regulation of disordered domains [8]. Dephosphorylated serine 17 (S17) favors Src dimerization and is found preferentially in cancer cells [9,10]. Phosphorylation of serine 75 (S75) causes changes in cell growth and cytoskeletal reorganization and mediates ubiquitination and degradation [11,12]. Phosphorylation of threonine 37 (T37) activates Src by disrupting the interaction between the SH2 domain and regulatory phosphotyrosine 530 [13]. Phosphorylation of serine 43 (S43) and serine 51 (S51) by Wnt3A have the opposite effects on Src activation [14].

Understanding the distinct and specific effects of phosphorylation at each of the sites starts by determining the functional unit that is affected by the introduction of the phosphate group. Is it strictly the modified residue or are there neighbor residues specifically affected, thus defining an extended functional site? Basic residues are obvious candidates to extend the functional effects of phosphorylation through interaction of the positively charged side chains with the negatively charged phosphate. Interestingly, in the Unique domain of c-Src, five of the functionally important serine and threonine phosphorylation sites have basic residues located three or less residues away along the sequence: S17 follows a stretch of three arginine residues, S75 is three residues away from arginine 78 (R78), lysine 40 (K40) is located between threonine 37 (T37) and serine 43 (S43), and S51 is three residues away from an arginine residue. 

To test the formation of specific contacts between phosphorylated serine or threonine residues and proximal basic groups, we have measured the changes in phosphate pKa caused by the mutation of individual basic groups. We show that the mere presence of a basic group in the proximity of the phosphate group does not imply the two groups are interacting. Additionally, by observing the effect of pH on the NH residues of wild-type phosphorylated c-Src, we can identify the ensemble of residues that are directly or indirectly affected by the titration of the phosphorylated residues. By comparing the pH dependency of NH chemical shifts in wild-type c-Src with the results obtained with mutated forms, we demonstrate a very simple NMR method to identify the phosphorylated residues that are interacting with basic residues that does not require mutagenesis or complete pKa determination. 

The Unique, SH4, and SH3 domains form the Src N-terminal regulatory element (SNRE), characterized by a fuzzy complex in which the Unique and SH4 domains form multiple, weak, dynamic contacts with the SH3 domain while remaining disordered. The SH3 domain couples with the other globular domains, presumably transmitting signaling events, such as phosphorylation of the disordered region to the kinase domain [15,16]. Electrostatic interactions are an important component of the fuzzy complex that can be modulated by phosphorylation [17]. The number and distribution of charged residues has been recognized as an important parameter affecting the conformational ensemble sampled by disordered protein regions [18]. Not surprisingly, phosphorylation of the Unique domain affects the fuzzy complex and the pKa of the phosphorylated residues are sensitive probes of this interaction.

We have centered this study on the sites that are phosphorylated by the mitogen activated kinase ERK2, a promiscuous kinase that preferentially phosphorylates serine or threonine residues followed by proline [19]. Unexpectedly, we found that ERK2 phosphorylates not only T37 and S75, but also S43, and to a minor extent, S51, although S43 and S51 are not followed by proline. 

## 2. Results

### 2.1. Phosphorylation of the Unique Domain of c-Src by ERK2

**Figure 1:**
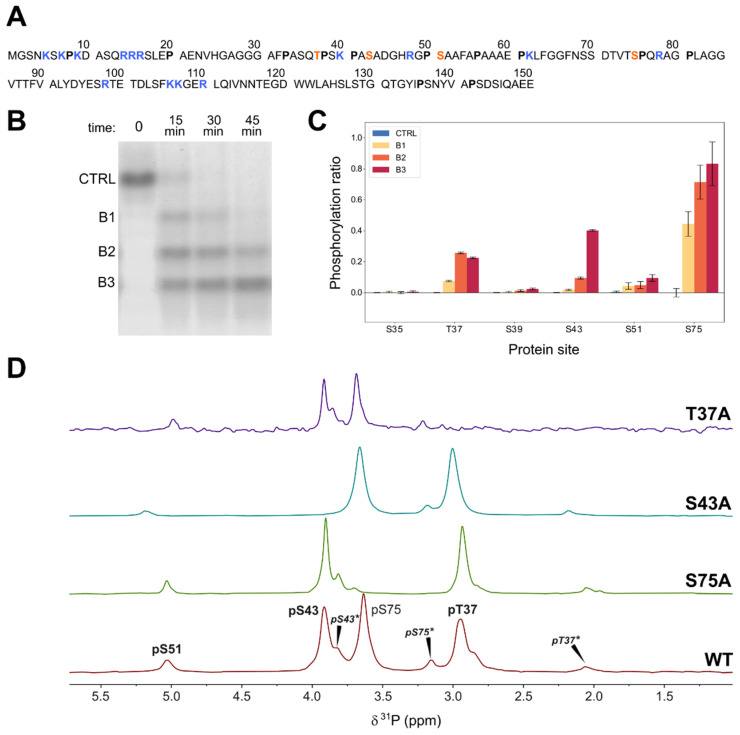
(**A**) Sequence of the N-terminal region of human c-Src constructs (USrc: 1-85; USH3: 1-150) with the phosphorylated residues studied in this work highlighted in red, basic residues marked in blue, and proline residues in bold face. (**B**) Native polyacrylamide electrophoresis during the phosphorylation of USH3 with ERK2. Bands are separated by the number of incorporated phosphate groups. (**C**) Phosphorylated sites detected by MS/MS in the three electrophoretic bands corresponding to the global incorporation of one, two, and three phosphates in USH3. The pS75 site is located in a different peptide than the other sites. (**D**) ^31^P-NMR (164 MHz) of wild-type USH3 and mutants used for NMR assignment. Asterisks denote peaks from forms containing cis proline bonds. The spectra were recorded in 50 mM 2-(N-morpholino) ethanesulfonic acid (MES) at pH 6.3 and referenced to triethylphosphate at 0.44 ppm.

The main phosphorylation sites in the Unique domain of human c-Src are T37 and S75, both of which are followed by proline residues. The S/TP motif is the consensus sequence for mitogen-activated kinases. We chose ERK2 for the in vitro phosphorylation of these two residues because of the availability of a constitutively activated ERK2 form [20]. Surprisingly, native gel electrophoresis of a construct including the SH4, Unique, and SH3 domains of c-Src (USH3) treated with ATP and ERK2 gave three major bands corresponding to the incorporation of up to three phosphate groups (Figure 1), suggesting the presence of at least an additional ERK2 target site besides T37 and S75. 

A tandem mass spectrometry analysis of the tryptic peptide that contains the T37 site confirmed that S43 was extensively phosphorylated by ERK2. An additional minor phosphorylation site, S51, was detected by mass spectrometry (Figure 1). These sites do not contain a canonical ERK2 substrate motif. Phosphorylation of S43 was confirmed by ^31^P-NMR (vide infra). Minor signals assigned to phosphorylated S51 were also detected by ^31^P-NMR and in some ^1^H-^15^N correlation spectra and confirmed by sequential NMR assignment in ^15^N and ^13^C labeled samples.

### 2.2. ^31^P-NMR

Figure 1 shows the ^31^P-NMR spectra of triple phosphorylated USH3 at pH 6.3 and 298K. The peaks of pS75 and pS43 overlap at pH 7. The three main peaks were assigned by comparison with mutated constructs in which the individual serine and threonine phosphosites were replaced by alanine. The disappearance of the signal at around 4.0 ppm in the S43A mutant confirmed the phosphorylation of S43 by ERK2. Additional low intensity peaks disappeared in each of the mutants, suggesting that they arise from the coexistence of phosphorylated peptides with a cis proline bond. The presence of cis proline forms was confirmed in ^13^C- and ^15^N-enriched samples (vide infra).

pT37 and pS75 that were located next to proline residues showed the largest upfield shifts when the proline adopts a cis conformation (ca. 0.9 ppm for T37 and 0.5 ppm for S75). pS43, which does have a direct proline neighbor, exhibits two minor forms at 0.1 and 0.2 ppm upfield, with relative integrals of 0.1 and 0.01 that suggest that pS43 is experiencing the effect of two different cis proline bonds.

The minor signal at around 5 ppm that is present in all the mutants was tentatively assigned to a small amount of phosphorylated serine 51 also detected by mass spectrometry. Consistent with this assignment, the chemical shift of this minor signal is most affected by the S43A mutation, which is the one closest to S51.

The pH dependence of the ^31^P-NMR signals was used to determine the apparent pKa values of the individual phosphate groups in the triple phosphorylated form of USH3 at 298K. The relevant pKa values were extracted by non-linear fitting of the Henderson–Hasselbalch equation. The pKa values of pT37, pS43, and pS75 are 6.10 ± 0.01, 5.80 ± 0.01, and 6.03 ± 0.01, respectively (Table 1). 

**Figure 2:**
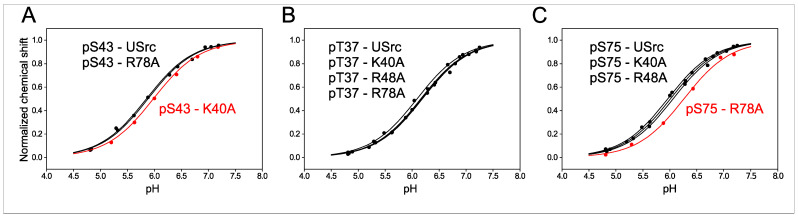
Normalized ^31^P-NMR chemical shift changes of (**A**) pS43, (**B**) pT37, and (**C**) pS75 phosphoresidues in USrc and single basic residue mutants. pKa shifts indicating the release of the interaction between pS43 and K40 and between pS75 and R78 are highlighted in red.

The pKa of the triple phosphorylated protein was also measured in USrc (Figure 2). The pKa values of pT37 and pS43 in the absence of the SH3 domain are 6.19 ± 0.01 and 5.88 ± 0.01, respectively, an increase of around 0.08 units. The pKa of pS75 shows a smaller increase of only 0.03 units, although S75 is closer in the sequence to the SH3 domain. This observation is compatible with the hypothesis that the pKa differences in the presence and in the absence of the SH3 domain are caused by changes in the fuzzy complex. 

To study the effect of the basic residues, we measured the pKa values of phosphate groups when residues K40, R48, and R78 were individually mutated to alanine in USrc (Figure 2). Phosphorylation of the R48A mutant produced only a double phosphorylated species containing pT37 and pS75, with S43 not phosphorylated.

Replacing the positively charged K40 by alanine caused a shift of +0.10 units in the pKa of pS43 (Table 1 and Figure 2A), strongly suggesting the formation of a salt bridge between the side chain of K40 and the phosphate group of pS43 that increases its acidity. Interestingly, the K40A mutation had no effect on the pKa of pT37 (Table 1 and Figure 2B), although the distances of K40 to T37 and S43 along the sequence are the same. The very different effect of the K40A mutation on these phosphate groups strongly suggests a direct interaction between the side chains of K40 and the phosphate group of pS43, as the electrostatic field generated by the positive charge in the lysine side chain is expected to be sensed similarly by the phosphate groups of pT37 and pS43. 

The pKa of pS75 experiences a large shift (+0.20 units) when R78 is mutated to alanine (Table 1 and Figure 2C), also suggesting the formation of a salt bridge between the arginine side chain and the phosphate group. 

The K40A mutation has a significant (−0.05 units) long-range effect on pS75 although in the opposite direction to that caused in pS43 (Table 1). This is an indirect effect probably caused by changes in the average distance of pS75 to the region including T37, K40, and S43. Long distance effects were observed in unphosphorylated USrc by paramagnetic relaxation experiments [21]. These long-range interactions also exist in the fuzzy complex with the SH3 domain. 

### 2.3. ^1^H-^15^N HSQC

**Figure 3:**
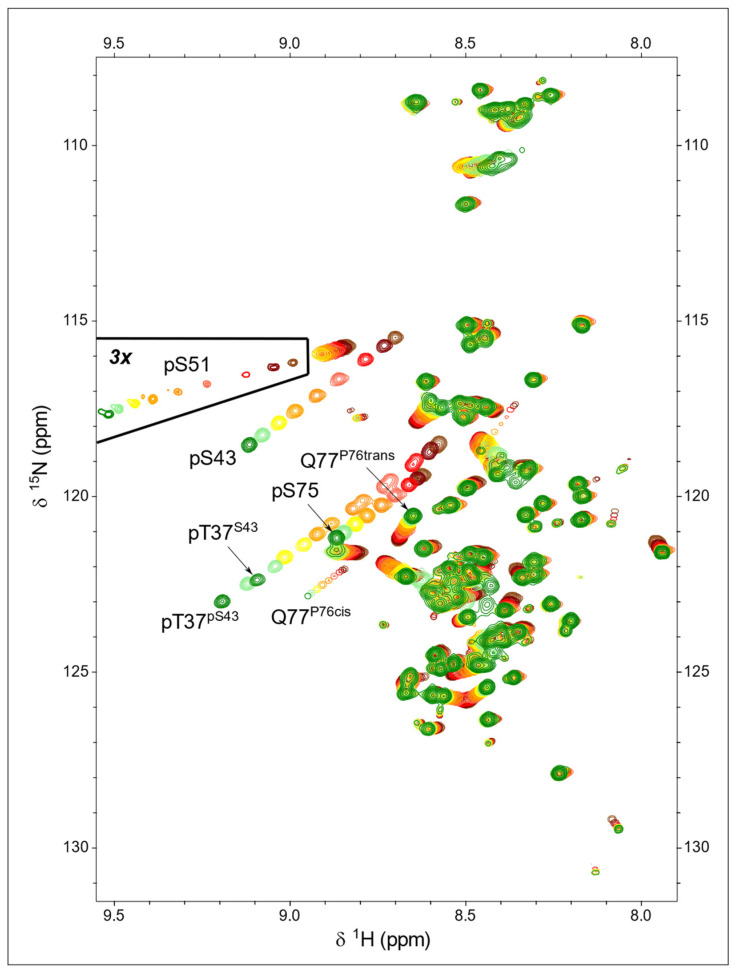
pH dependency of ^1^H-^15^N-HSQC spectra of ERK2-phosphorylated USrc between pH 7.2 (green) and pH 4.7 (brown). The peaks showing the largest pH dependency are those of the backbone NH of the phosphorylated residues. The marked region was plotted at a lower contour level to show the small quantity of phosphorylated S51 present in this sample.

Phosphorylation of serine and threonine residues caused a very large shift in the NH peak of the phosphorylated residue. ^1^H-^15^N-HSQC experiments of USH3 and USrc phosphorylated with ERK2 show three main peaks in the region corresponding to phosphorylated serine and threonine, all of which exhibit a strong pH dependency (Figure 3). This contrasts with previous reports showing that only T37 and S75 were phosphorylated by CDK5/p25 [21]. The new peak was assigned to pS43, confirming the mass spectrometry results. 

The NH signals from the three phosphorylated residues, pT37, pS43, and pS75, shifted downfield significantly in the proton and nitrogen dimensions with a very similar slope on transitioning from the predominantly single-protonated (i.e., monoanionic phosphate) form at pH 4.7 to the unprotonated (i.e., dianionic phosphate) form at pH 7.2. Thus, the corresponding peaks move following parallel lines (Figure 3).

A weak signal in the HSQC spectrum of USrc could be unequivocally assigned to the small amount of phosphorylated S51, and the pKa of this minor form could be determined to be 5.61 ± 0.01. The phosphate group in pS51 is more acidic than those of the other serine residues in USrc. The small signal at low field in the ^31^P-NMR spectra of the K40A mutant USrc, which gave the strongest signal, has an apparent pKa of 5.72, consistent with the tentative assignment of this signal to pS51. 

Phosphorylation also affects the NH chemical shifts of residues other than the phosphorylated ones. In those residues, the relative effects of pH on the ^1^H and ^15^N dimensions may be different to those of the phosphorylated residues; thus, the peaks follow lines with different slopes. The direction of the peak displacement reflects the combination of various types of interactions, such as electrostatic effects, hydrogen bonding, or conformational shifts, that may differently affect the two nuclei. 

**Figure 4:**
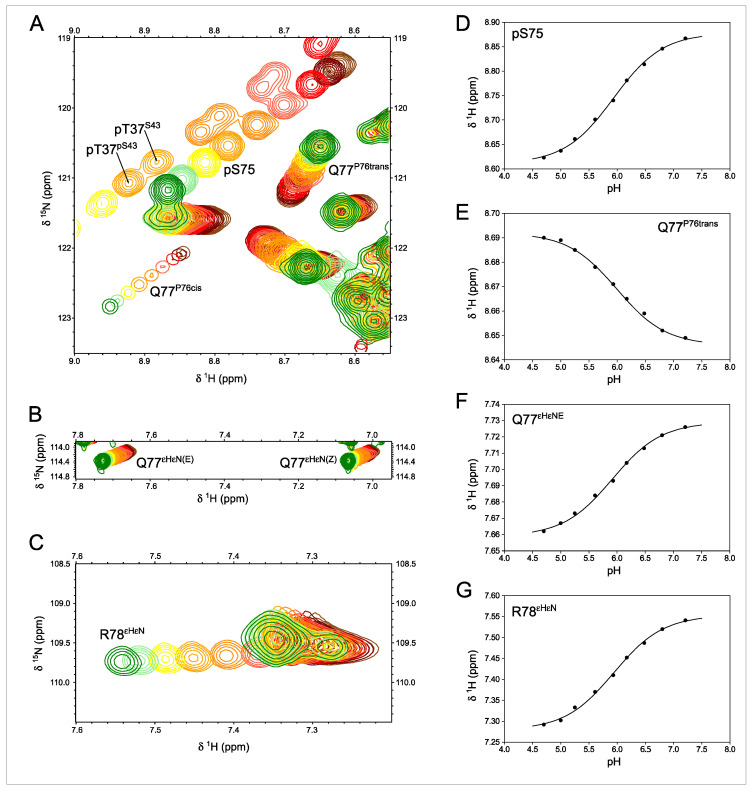
^1^H-^15^N-HSQC-monitored pH titration of ERK2-phosphorylated Usrc. (**A**–**C**) Expansions of spectra showing the effect of titrating the phosphate groups between pH 7.2 (green) and pH 4.7 (brown). Labeled peaks are those affected by the titration of pS75, except pT37. The R78 side chains shown in C are folded in the ^15^N dimension. (**D**–**G**) Various peaks affected by the titration of pS75 show the same pKa value. The identical pKa values measured in pS75 backbone NH and the side chains of R78 and Q77 confirm the direct interaction between pS75 and R78. The two sets of peaks for pT37 arise from the coexistence in this sample of species with phosphorylated and unphosphorylated S43 that have distinct pT37 pKa values.

The pKa measured from these signals can be used to identify the titrating group primarily responsible for the observed pH dependency. This is the case for the NHε of the guanidinium side chain of R78 that titrates with a pKa of 5.94 ± 0.01, identical, within the experimental error, to that measured in the NH of pS75 (5.93 ± 0.01), confirming the direct interaction between pS75 and R78 side chains. The carboxamide NH_2_ signals and backbone NH of Q77 are also titrated with the same apparent pKa (5.92 ± 0.02) (Figure 4).

The HSQC experiments reveal additional peaks that could be assigned to residues next to proline in the cis conformation. The configuration of the proline peptide bond could be determined from the difference between the chemical shifts of the β and γ carbons, which is around 10 ppm in the cis form and only 5 ppm in the trans configuration [22]. The pH-induced chemical shifts of Q77 NH in the major trans form go in the opposite direction of those of pS75 (Figure 4D,E), suggesting an indirect effect affecting the Q77 side chain when the phosphate group of pS75 and the guanidinium group of R78 interact.

The NH main chain signal of Q77 in the species with a *cis* and *trans* peptide bond at proline 76 is well resolved. The pH-induced chemical shift changes of the NH of Q77 in the cis form follow a similar slope to those of pS75 (Figure 3), suggesting that the interaction between the phosphate and the pS75 and Q77_(P76 cis)_ backbone NH are similar, which is compatible with the proximity of Q77 and S75 when the intervening proline is in the cis form.

Phosphorylation of S43 by ERK2 is slower than that of T37 and S75 that contain the consensus target sequence. Thus, it was possible to prepare samples that contained a mixture of phosphorylated and non-phosphorylated S43 that could be easily distinguished in the HSQC experiments. This allowed to determine the effect of the phosphate group in S43 on the pKa of pS37 (Figure 4A); the presence of the negatively charged pS43 makes the phosphate group of pT37 less acidic by +0.07 units.

### 2.4. Formation of a Fuzzy Complex Affects the pKa of Phosphorylated Residues

The pKa values of pT37 and pS43 measured at 298K in the presence of the SH3 domain are 0.09 and 0.08 units lower, respectively, than those measured in the isolated disordered region (Table 1). The difference is smaller for pS75 (−0.03 units). The differences observed at 278K follow the opposite trend; the pKa values increase when the SH3 domain is present, and the largest changes are observed for pS75 (+0.06) and pS43 (+0.06) and the pKa of pT37 shifts by +0.03 units (Table 2). All the changes are significant and suggest the electrostatic environment of the phosphorylated groups is affected by long-range interactions in the fuzzy complex formed between the disordered and SH3 domains.

The observation that the apparent acidity of the phosphate groups in the fuzzy complex [23], as compared to those in the isolated disordered domain, increases at high temperature and decreases at 278K is interesting and indicates the complexity of the multiple competing interactions present in the fuzzy complex. The importance of electrostatics in fuzzy intermolecular complexes is well recognized [24]

The pH of phosphate buffer decreases by approximately 0.003 pH units per degree [25]. The observed pKa change from 278 K to 298 K of pT37 and pS43 in pUSH3 (−0.06 units) agrees with this expectation, but the pKa of pS75 changes in the opposite direction. The pKa of phosphate groups in the isolated disordered domain increases with temperature. We can only speculate on the origin of the opposite signs of the pKa temperature coefficients in the fuzzy complex and the isolated disordered region or the opposite trend observed in the pKa changes caused by the presence of the SH3 domain at 278 K and 298 K. The SH3 domain has a global negative charge, although it contains both positively and negatively charged residues. The fact that the pKa temperature coefficients of p37 and p43 in pUSH3 are similar to that of an isolated phosphate group may suggest that the negative SH3 is competing with the phosphate group for the interaction with the positively charged groups. Additionally, the decreased apparent acidity observed at the lower temperature is consistent with the overall expected effect of the phosphate groups being close to the negatively charged SH3 domain.

In the absence of the SH3 domain, the interaction between the phosphate group and nearby positively charged groups is unhindered and it may cause the pKa temperature coefficients to be opposite in sign to those of an isolated phosphate group. Interestingly, the phosphate group of pS75, which shows the strongest basic residue effects on its pKa, is the one exhibiting more extreme temperature coefficients in pUSrc and the only one with a positive coefficient in pUSH3. The opposite effects of the SH3 domain on the pKa of phosphorylated residues at low and high temperatures may reflect changes in the compaction of the fuzzy complex or a preferential interaction of the phosphate groups with positively charged residues in the SH3 domain, such as the key arginine residue located in the RT loop of the SH3 domain of Src.

### 2.5. A Fast Method to Identify Free or Interacting Phosphate Groups Directly from the pH Dependency of HSQC Spectra

The magnitude of the pH-induced chemical shift change provides a direct indication of whether the phosphate group is interacting with distant sites, such as basic residues, or not.

**Figure 5:**
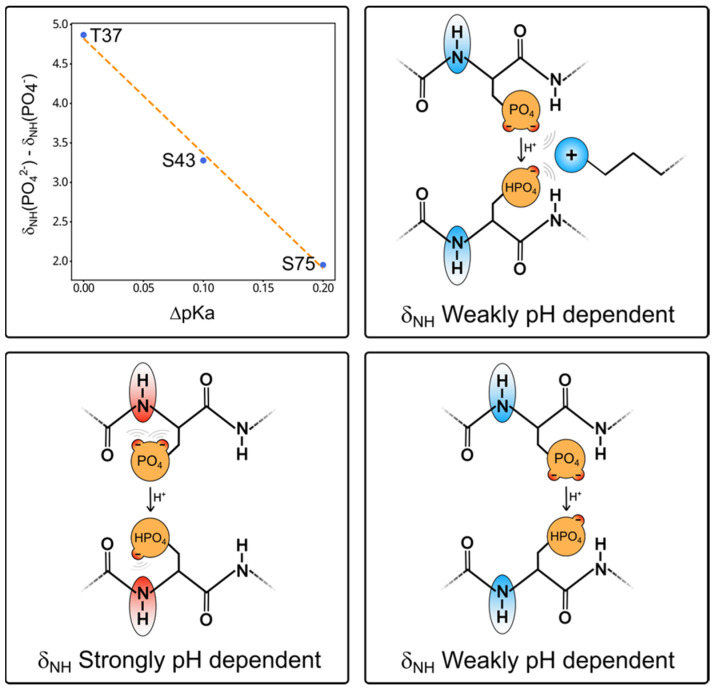
The top left graph shows the linear dependency between the change in pKa induced by the mutation of the nearest basic residue (K40 for T37 and S43; and R78 for S75) and the nitrogen chemical shift difference between the two ionization states of the phosphate group extracted from the fitting of the Henderson–Hasselbalch equation. The cartoons illustrate the origin of the pH dependency of the backbone NH groups of phosphorylated serine and threonine residues. In the bottom panel, two conformations placing the phosphate group close (**left**) or far from (**right**) the NH group are indicated. The ionization state of the phosphate group has a maximum effect on the NH chemical shift when it is close. This would be the preferred conformation due to hydrogen bonding between the phosphate and the peptide backbone. In the top cartoon, the basic side chain interacts with the phosphate and moves it away from the peptide backbone, thus making the NH chemical shifts less pH dependent. Thus, by simply comparing the NH chemical shifts of a phosphorylated residue at pH values well above and below the pKa is enough to determine if the phosphate group is free or interacting.

The difference between the high and low pH chemical shifts of the NH of pS75, pS43, and pT37 shows a nearly linear correlation with the pKa shifts experienced by the respective phosphate groups when the interacting basic residue was mutated to alanine (Figure 5).

The phosphate groups of phosphoserine and phosphothreonine can form a hydrogen bond with the NH of the same residue. This interaction is strongly dependent on the ionization state of the phosphate group, so the NH peak experiences large pH-dependent shifts. When the phosphate group participates in a competing external interaction (i.e., with a close basic group), its interaction with the NH group is weakened and the pH-dependent shift is smaller. Thus, recording two HSQC spectra above and below the expected pKa of the phosphate group and measuring the chemical shift change enables the prediction of whether the phosphate group is free or interacting strongly.

## 3. Discussion

Phosphorylation is a key post-translational modification through which cell signaling is transmitted. These modifications frequently occur in disordered regions, but their effects are transmitted to the entire protein. The Src N-terminal regulatory element (SNRE) reads the cellular environment through the disordered Unique and SH4 domains to determine the precise outcome of downstream signaling. c-Src is a non-receptor tyrosine protein kinase at the crossroads of many signaling pathways. The Unique domain contains two canonical phosphosites targeted by mitogen-activated protein kinases (MAPK). NMR and mass spectrometry results show that at least two additional sites are phosphorylated in vitro by MAPK1, also known as extracellular regulated kinase (ERK2). All these sites have a basic residue located three residues upstream or downstream along the sequence.

The pKa of each of the phosphosites has been determined in the isolated disordered region and in the entire SNRE, including the globular SH3 domain, in addition to the disordered domains. Our results clearly show that arginine 78 has a direct interaction with the phosphate group of pS75 that induces a shift in its pKa of around 0.2 units. Lysine 40 interacts with pS43, resulting in a pKa shift of around 0.1 units. pT37, despite having a lysine residue at position i + 3, is not affected by the mutation of K40.

Phosphorylation of the Unique domain of c-Src by ERK2 causes smaller but significant pH-dependent chemical shift perturbations in distant residues along the sequence. The pKa of the group causing the observed shifts can be used to identify the specific phosphate group responsible for the perturbation. The effects observed in the guanidinium side chain of R78 and in the intervening residue Q77 provide additional evidence of the direct interaction between pS75 and R78.

In this work, we have used point mutations combined with NMR-based pKa measurements to identify interactions affecting the phosphorylated residues in the Unique domain. These are time-consuming experiments. However, our data suggest a much simpler approach to identifying phosphate groups that have a strong interaction with a basic group; the magnitude of the chemical shift difference in the HN group of the phosphorylated residues correlates inversely with the intensity of the interaction of the phosphate group with neighbor basic residues, as determined by the pKa shift induced when the interacting basic residue is mutated. Thus, by measuring ^1^H-^15^N correlation spectra of a protein with phosphorylated serine or threonine residues at two pH values (e.g., pH 5.0 and pH 7.5), encompassing the expected pKa of the phosphate group and observing the pH-induced shift, one can identify if a particular phosphate group is free or interacting with a neighbor basic group. ^15^N chemical shifts changes of phosphorylated serine or threonine residues that are not interacting with a basic group have a large pH dependency (on the order of 4–5 ppm), while those with a strongly interacting phosphate show a much smaller dependency (1.5–2 ppm). Thus, a simple preliminary experiment at two pH values provides evidence of the interaction between the phosphate group and basic residues as well as an estimate of the strength of this interaction. Final confirmation will require the complete pKa determination in the wild-type protein as well as basic residue point mutants as described in this paper.

This simple approach has some analogy with the classical identification of intramolecular hydrogen bonds in peptides or proteins through the measurement of amide proton temperature coefficients. In our proposed approach, instead of changing the temperature, we change the pH. Additionally, similar to the use of temperature coefficients, the presence of the relevant interactions causes a smaller sensitivity to the external perturbation (low temperature coefficients and small pH dependency of NH chemical shifts). This is due to the competition of the relevant interaction, which has a small dependency on temperature or pH, with other trivial interactions that have a higher sensitivity to perturbations.

In phosphorylated serine and threonine residues, the phosphate group strongly interacts with the NH group of the phosphorylated residue and the resulting shift in the NH group is strongly pH dependent, as it is directly affected by the ionization state of the phosphate group (Figure 4). When the phosphate group is interacting with a neighbor basic residue, changes in its ionization state have only a weak effect on the NH group of the phosphorylated residue. Thus, the interaction of the phosphate with an external basic group reduces the pH dependency of the NH chemical shifts. It should be clear that this effect applies to phosphorylated serine and threonine residues but not to phosphotyrosine, whose phosphate group cannot interact directly with the backbone NH of the same residue.

## 4. Materials and Methods

*Cloning, Prokaryotic Gene Expression, and Protein Production.* Wild-type USrc encompassing residues 1–88 of c-Src with a C-terminal streptag and USH3 including residues 1–150 with an N-terminal His_6_-GST fusion have been previously described [23]. The production protocol was modified by using an N-terminal His-tagged SUMO fusion protein that enabled affinity purification followed by cleavage with Ubiquitin-like-specific protease 1 (ULP1) that does not leave non-natural residues at the extremes. In the case of the USrc construct, a non-natural tyrosine was introduced at position 86 near the C-terminus to facilitate detection and quantification by UV.

Forward and reverse primers were designed to create point mutants at phosphosites (T37A, S43A, and S75A) or at basic residues (K40A, R48A, and R78A). QuikChange II XL, a PCR site directed mutagenesis kit (Agilent Technologies), was used according to the instruction manual for mutagenesis PCR.

pET28b plasmids encoding His-tagged SUMO at the 5′-end of the corresponding construct were cloned into Omnimax cells and verified by sequencing. The correct plasmids were expressed in *E. coli* BL21 (DE3) pLysS Rosetta cells exploiting kanamycin and chloramphenicol resistances. Cells were grown in Luria Broth medium for unlabeled samples or minimal M9 medium with ^15^NH_4_Cl or ^15^NH_4_Cl, and [U-^13^C]-glucose for isotopically labeled samples until OD600 reached 0.7. Protein expression was induced by 200 µM isopropyl-β-D-1-thiogalactoside (IPTG) at 25 °C overnight. After induction, cells were centrifuged at 5000× *g* for 20 min and the pellet was resuspended in lysis buffer (50 mM Na-Phosphate, 300 mM NaCl, pH 8.0) containing phenyl-methyl-sulfonyl-fluoride (PMSF) and benzamidine protease inhibitors both at 1 mM, and either kept at −80 °C for later use or immediately lysed in the presence of 20 mM imidazole, lysozyme (625 μg/mL), and DNase I (10 μg/mL) by sonication in an ice bath. Cell debris and liquid lysate were separated for 25 min at 20 000 rpm (48,380× *g*). The supernatant was loaded into a NiNTA affinity column (5 mL), washed with lysis buffer, and the desired protein was eluted with 400 mM imidazole. The eluate was dialyzed against 50 mM Na-Phosphate, 300 mM NaCl, pH 8.0, and 0.5–1.0 mM DTT using a 3.5–5 kDa cut-off bag in the presence of ULP1 for at least two hours to remove the excess of imidazole and cleave N-terminal His-tagged SUMO, which was subsequently removed by a second NiNTA column. The flow through was injected in a preparative size exclusion S75 column (approx. 180 mL). In a few instances, anion exchange chromatography was used before gel filtration. The fractions of gel filtration were checked on 16% SDS-PAGE and the fractions containing pure protein were concentrated to about 700 µM.

*ERK2 production and phosphorylation reactions.* A plasmid encoding a constitutively active, His-tagged form of ERK2 was kindly provided by Prof. Attila Reményi [24] and expressed according to the protocol provided by this group. Briefly, the protein was expressed in *E. coli* BL21 Rosetta (DE3) grown in Terrific Broth medium. Protein induction was carried out with 0.3 mM IPTG when the OD_600_ of the culture reached 0.3–0.4. Following lysis by sonication, the soluble fraction was purified by nickel affinity using a 5 mL His Trap FF column, eluted with 400 mM imidazole, and further purified by size exclusion using a Sephadex S75 column in 50 mM Tris, 50 mM NaCl, pH 7.4 buffer containing 10% glycerol and 0.01% sodium azide. The purified protein was aliquoted (150 μM, 210 μL), quickly frozen in liquid nitrogen, and stored at −80 °C until it was used.

Phosphorylation reactions were carried out at a ERK2 to substrate ratio of 1:50. The reaction conditions were 50 mM phosphate buffer pH 7.4, 5 mM ATP, 10 mM MgCl_2_, 1 mM dithiothreitol (DTT), 3 μM ERK2, and 150 μM substrate. Complete conversion was achieved after three hours at room temperature (approx. 20 °C). USH3 samples were purified by removing ERK2 using a nickel column followed by size exclusion chromatography. The USrc and mutated samples were directly dialyzed against 50 mM citrate buffer (pH 7.2) and concentrated to 70–200 μM.

*Identification of phosphorylation sites by mass spectrometry.* A sample of USH3 phosphorylated by ERK2 gave three distinct bands in native 8% polyacrylamide gel electrophoresis, corresponding to species with a different number of phosphorylation sites, with no band corresponding to the non-phosphorylated form. The three bands were independently excised from the gel, destained with NH_4_HCO_3_ and acetonitrile, reduced with 10 mM DTT for 45 min at 56 °C, and alkylated with 50 mM iodoacetic acid in the dark. Following this treatment, the bands were digested with trypsin (sequencing grade modified trypsin, Proomega Cat#V511) at 37 °C overnight. The digestion was stopped with 5% formic acid and the peptides were eluted with acetonitrile. The sample solution was dried completely in SpeedVac and reconstituted in 20 μL of a 3% acetonitrile/1% formic acid aqueous solution for mass spectrometry analysis.

Tryptic digested peptides were trapped with a μ-precolumn (300 μm i.d. × 5 mm PepMap100, 5 μm, 100 Å, C18 (Thermo Scientific)) and separated by a C18 analytical column NanoEase MZ HSS T3 column (75 μm × 250 mm, 1,8 μm, 100 Å) (Waters). The column outlet was directly connected to an Advion TriVersa NanoMate (Advion) fitted on an Orbitrap Fusion Lumos Tribid (Thermo Scientific, Waltham, MA, USA). Tandem mass spectrometry (MS/MS) was carried out using a higher energy collisional dissociation (HCD). We performed a twin database search with Thermo Proteome Discoverer v2.5.0.400 (PD) and MaxQuant v1.6.17.0 (MQ) using the search engines Sequest HT for PD and Andromeda for MQ. The databases used were Human and *E. coli* SwissProt release 01/2021 and the common contaminants database as well as the Decoy database to discover the false discovery rate. The search parameters included trypsin enzyme specificity, allowing for two missed cleavage sites, oxidation of methionine, phosphorylation of serine, threonine, and tyrosine, and acetylation in the protein N-terminus as dynamic modifications. The ptmRS node of PD was used to provide a confidence measure for the localization of phosphorylations. Phosphopeptide spectrum matches containing any phosphorylation sites with localization probability <75% were tagged as ambiguous. For unambiguous sites found with more than one peptide spectrum match (PSM), we computed the phosphorylation ratio (r) for a phosphorylation site from the maximum number of PSMs for phosphorylated (N_Phos_) and non-phosphorylated (N_NoPhos_) peptides identified by the Sequest HT and Andromeda nodes. The ratio was computed as r = N_Phos_/(N_Phos_ + N_NoPhos_).

*^31^P NMR measurements and data analysis.* NMR measurements were conducted on a Bruker Avance III operating at 9.4 T (400 MHz for ^1^H and 162 MHz for ^31^P) equipped with a sample exchanger. Unless otherwise stated, the samples were dissolved in 50 mM citrate buffer and the temperature was 298 K. Triethylphosphate was used as a reference. Spectra were processed and analyzed with Topspin 3.4 and MestreLab Research S.L. (analysis).

*pH titration and pKa calculations.* For ^31^P-NMR using unlabeled proteins*,* separate samples were adjusted at the desired pH values in the range between 4.8 and 7.2. For isotopically labeled samples, the pH was adjusted between experiments using concentrated HCl or NaOH.

pKa values were determined by non-linear fitting to the Henderson–Hasselbalch equation, leaving pKa and the high and low pH chemical shifts as adjustable values. The accuracy of the pKa was estimated by Monte Carlo fitting using a collection of 10 artificial datasets generated by adding normal distributed errors of 0.01 ppm to the measured chemical shifts and 0.01 units to the pH measurements of the experimental data points and independently fitting each set to obtain a set of pKa values from which the mean and standard deviations were calculated.

*2D and 3D NMR experiments.* ^1^H-^15^N-HSQC experiments were recorded at 278K in 600 MHz Bruker Avance III or Bruker 800 MHz Avance Neo instruments equipped with TCI cryoprobes. Backbone NH assignments of double-labeled (^13^C-^15^N) USH3 and pUSH3 were confirmed by 3D experiments and transferred to pUSrc. The measuring time was shortened by using best-TROSY versions and a non-uniform sampling strategy (25% NUS) of the following experiments: HNCA, HN(CO)CA, HNCACB, HN(CO)CACB, HNCO, HN(CA)CO, and (H)CC(CO)NH-TOCSY at 800 MHz*. Cis* and *trans* proline residues were assigned based on the CB and CG chemical shift differences obtained from (H)CC(CO)NH spectra. Sidechain assignments of Q77 were based on modified HNCACB experiments. For ε-NH Arg, we used the HiSQC approach [26] and connection to the other side chain protons through 2D HCN(CC)H-TOCSY and 3D TOCSY-HSQC ^1^H-^15^N (t_mix_ = 80 ms) as well as 3D NOESY-HSQC ^1^H-^15^N (t_mix_ = 120 ms). R78 ε-NH showed clear TOCSY transfer along its side chain. R48 gave weaker connections but showed confirmed NOEs from HA, HB, HG, and HD protons to G49 NH.

## 5. Conclusions and Future Perspectives

The pKa values of phosphorylated residues are affected by short- and long-range electrostatic interactions and provide a sensitive probe for the conformational ensemble sampled by disordered domains. pKa measurements at two temperatures and in the presence or absence of the SH3 domain acting as a scaffold of the intramolecular fuzzy complex adopted by the SNRE reveal a complex scenario. The balance between competing interactions provides a sensitive test to validate calculations of the conformational ensemble adopted by disordered domains [27], especially in the vicinity of SH3 domains [28], although this is beyond the scope of the present article.

Phosphorylation of intrinsically disordered domains are important regulatory events. Here we show that neighbor basic residues interacting with the phosphorylated groups may form multi-residue functional units and we provide a simple NMR test to identify and quantify the strength of the interaction of phosphate groups with neighbor residues. Relatively distant phosphosites may interact with each other through an intermediate basic group. This is probably the case of the opposite effects of phosphorylation of S43 and S51, coupled through R48, although the low phosphorylation yield of S51 by ERK2 has prevented a more detailed study. In the presence of multiple ionizable groups, proton exchange between distinct sites introduces yet another regulatory element, including an entropic component and the coupling between distinct charge states and conformational regulation [29].

## Figures and Tables

**Table 1:** pKa values in wild-type USH3, USrc, and basic residue mutants ^1^.

Phosphosite ^1^	USrc Wild Type	USrcK40A	USrcR48A ^3^	USrcR78A	USH3 Wild Type
pT37	6.19	6.18 (−0.01) ^2^	6.09 (−0.10)	6.16 (−0.03)	6.10 (−0.09)
pS43	5.88	5.98 **(+0.10)**	-	5.85 (−0.03)	5.80 (−0.08)
pS75	6.06	6.01 (−0.05)	5.96 (−0.10)	6.26 **(+0.20)**	6.03 (−0.03)

^1^—^31^P-NMR; T = 298 K. ^2^—Values in parentheses are the pKa difference with respect to wild-type phosphorylated USrc. Error limits estimated from a Monte-Carlo analysis are ± 0.01 (see experimental part). ^3^—Phosphorylated only at T37 and S75. pKa changes indicating direct interaction of the phosphate group with a neighbor basic group are highlighted in bold type.

**Table 2:** Temperature-dependent pKa in the fuzzy complex and isolated disordered domains.

Phosphosite ^1^	Temp. ^2^	pT37	pS43	pS75
pUSrc	278	6.13	5.80	5.93
pUSrc	298	6.19	5.88	6.06
pUSH3	278	6.16	5.86	5.99
pUSH3	298	6.10	5.80	6.03

^1^—Error limits in pKa estimated from Monte-Carlo analysis are ± 0.01 (see experimental part). ^2^—pKa values determined by ^31^P-NMR at 298K and by ^1^H-^15^N HSQC at 278K.

## Data Availability

Data are available from the corresponding author.

## Sample Availability

Plasmids to express the proteins are available from the authors.

## References

[1] Yeatman T.J. (2004). A renaissance of SRC. Nat. Rev. Cancer.

[2] Sirvent A., Benistant C., Roche S. (2012). Oncogenic signaling by tyrosine kinases of the SRC family in advanced colorectal cancer. Am. J. Cancer Res..

[3] Hynes N.E. (2000). Tyrosine kinase signalling in breast cancer. Breast Cancer Res..

[4] Xu W., Doshi A., Lei M., Eck M.J., Harrison S.C. (1999). Crystal structures of c-Src reveal features of its autoinhibitory mechanism. Mol. Cell.

[5] Cowan-Jacob S.W., Fendrich G., Manley P.W., Jahnke W., Fabbro D., Liebetanz J., Meyer T. (2005). The crystal structure of a c-Src complex in an active conformation suggests possible steps in c-Src activation. Structure.

[6] Pérez Y., Maffei M., Igea A., Gairí M., Nebreda A.R., Bernadó P., Pons M. (2013). Lipid binding by the Unique and SH3 domains of c-Src suggests a new regulatory mechanism. Sci. Rep..

[7] Aponte E., Lafitte M., Sirvent A., Simon V., Barbery M., Fourgous E., Boublik Y., Maffei M., Armand F., Hamelin R. (2022). Regulation of Src tumor activity by its N-terminal intrinsically disordered region. Oncogene.

[8] Amata I., Maffei M., Pons M. (2014). Phosphorylation of unique domains of Src family kinases. Front. Genet..

[9] Ruiz-Saenz A., Zahedi F., Peterson E., Yoo A., Dreyer C.A., Spassov D.S., Oses-Prieto J., Burlingame A., Moasser M.M. (2021). Proteomic analysis of Src family kinase phosphorylation states in cancer cells suggests deregulation of the unique domain. Mol. Cancer Res..

[10] Le Roux A.-L., Castro B., Garbacik E.T., Garcia Parajo M.F., Pons M. (2016). Single molecule fluorescence reveals dimerization of myristoylated Src N-terminal region on supported lipid bilayers. ChemistrySelect.

[11] Kato G., Maeda S. (1997). High-level expression of human c-Src can cause a spherical morphology without loss of anchorage-dependent growth of NIH 3T3 cells. FEBS Lett..

[12] Pan Q., Qiao F., Gao C., Norman B., Optican L., Zelenka P.S. (2011). Cdk5 targets active Src for ubiquitin-dependent degradation by phosphorylating Src(S75). Cell Mol. Life Sci..

[13] Stover D.R., Liebetanz J., Lydon B.N. (1994). Cdc2-mediated Modulation of pp60c-src activity. J. Biol. Chem..

[14] Kim J.G., Mahmud S., Min J.K., Lee Y.-B., Kim H., Kang D.-C., Park H.-S., Seong J., Park J.-B. (2020). RhoA GTPase phosphorylated at tyrosine 42 by src kinase binds to β-catenin and contributes transcriptional regulation of vimentin upon Wnt3A. Redox Biol..

[15] Maffei M., Arbesú M., Le Roux A.-L., Amata I., Roche S., Pons M. (2015). The SH3 domain acts as a scaffold for the N-terminal intrinsically disor-dered regions of c-Src. Structure.

[16] Arbesú M., Iruela G., Fuentes H., Teixeira J.M.C., Pons M. (2018). Intramolecular fuzzy interactions involving intrinsically disordered domains. Front. Mol. Biosci..

[17] Zhou J., Zhao S., Dunker A.K. (2018). Intrinsically Disordered Proteins Link Alternative Splicing and Post-translational Modifications to Complex Cell Signaling and Regulation. J. Mol. Biol..

[18] Das R.K., Pappu R.V. (2013). Conformations of intrinsically disordered proteins are influenced by linear sequence distributions of oppositely charged residues. Proc. Natl. Acad. Sci. USA.

[19] Zhang J., Zhang F., Ebert D., Cobb M.H., Goldsmith E.J. (1995). Activity of the MAP kinase ERK2 is controlled by a flexible surface loop. Structure.

[20] Alexa A., Sok P., Gross F., Albert K., Kobori E.W., Póti A.L., Gógl G., Bento I., Kuang E., Taylor S.S. (2022). A non-catalytic herpesviral protein reconfigures ERK-RSK signaling by targeting kinase docking systems in the host. Nat. Commun..

[21] Pérez Y., Gairí M., Pons M., Bernadó P. (2009). Structural Characterization of the Natively Unfolded N-Terminal Domain of Human c-Src Kinase. Insights into the Role of Phosphorylation of the Unique Domain. J. Mol. Biol..

[22] Schubert M., Labudde D., Oschkinat H., Schmieder P. (2002). A software tool for the prediction of Xaa-Pro peptide bond conformations in proteins based on ^13^C chemical shift statistics. J. Biomol. NMR.

[23] Arbesú M., Maffei M., Cordeiro T.N., Teixeira J.M., Pérez Y., Bernadó P., Roche S., Pons M. (2017). The Unique Domain Forms a Fuzzy Intramolecular Complex in Src Family Kinases. Structure.

[24] Fuxreiter M. (2022). Electrostatics tunes protein interactions to context. Proc. Natl. Acad. Sci. USA.

[25] Mohan C. (2006). Buffers: A Guide for The Preparation and Use of Buffers in Biological Systems.

[26] Iwahara J., Jung Y.-S., Clore M.G. (2007). Heteronuclear NMR spectroscopy for lysine NH_3_ groups in proteins:  unique effect of water exchange on ^15^N Transverse Relaxation. J. Am. Chem. Soc..

[27] Shrestha U.R., Smith J.C., Petridis L. (2021). Full structural ensembles of intrinsically disordered proteins from unbiased molecular dynamics simulations. Commun. Biol..

[28] Gurumoorthy V., Shrestha U.R., Zhang Q., Pingali S.V., Boder E.T., Urban V.S., Smith J.C., Petridis L., O’Neill H. (2023). Disordered Domain Shifts the Conformational Ensemble of the Folded Regulatory Domain of the Multidomain Oncoprotein c-Src. Biomacromolecules.

[29] Fossat M.J., Posey A.E., Pappu R.V. (2021). Quantifying charge state heterogeneity for proteins with multiple ionizable residues. Biophysical J..

